# Supplementary material for: Tuning intrinsic disorder predictors for virus proteins
Source: Virus Evol. 2021 Jan 25;7(1):veaa106. doi: 10.1093/ve/veaa106 (PMC7882063; doi:10.1093/ve/veaa106)
Supplement: veaa106_Supplementary_Data [file veaa106_supplementary_data.pdf]

Supplementary Tables

Table S1: Summary of viral protein data

Family	Organism	Protein	Disprot ID	Length
Alphafusellovirus	Sulfolobus spindle-shape virus 1	Protein F-112	DP00847	112
Alphatectivirus	Enterobacteria phage PRD1	Protein P16	DP01012	117
Betapolyomavirus	JC polyomavirus	Agnoprotein	DP01186	71
	Simian virus 40	Large T antigen	DP01618	708
		Major capsid protein VP1	DP00182	362
Chordopoxvirinae	Molluscum contagiosum virus subtype 1	Viral CASP8 and FADD-like apoptosis regulator	DP02042	241
	Myxoma virus	M156R	DP00849	102
		Probable host range protein 2-3	DP01983	203
	Vaccinia virus	Protein F1	DP01539	222
		Protein K7	DP02194	149
Deltavirus	Hepatitis delta virus genotype I	Small delta antigen	DP00965	195
Firstpapillomavirinae	Human papillomavirus type 16	Protein E6	DP01615	158
		Protein E7	DP00024	98
		Regulatory protein E2	DP01428	365
	Human papillomavirus type 45	Protein E7	DP01780	106
		Protein E7	DP00947	106
	Human papillomavirus type 51	Protein E6	DP02256	151
Flaviviridae	Bovine viral diarrhea virus	Genome polyprotein	DP00675	3988
	Dengue virus type 1	Genome polyprotein	DP01929	3392
	Dengue virus type 2	Genome polyprotein	DP01930	3391
			DP01245	3388
			DP00876	3391
	Dengue virus type 3	Genome polyprotein	DP02204	3390
	Dengue virus type 4	Genome polyprotein	DP01931	3387
	Hepatitis C virus genotype 1a	Genome polyprotein	DP00588	3011
	Hepatitis C virus genotype 1b	Genome polyprotein	DP01142	3010

Table S1: Summary of viral protein data

Family	Organism	Protein	Disprot ID	Length
Flaviviridae	Hepatitis C virus genotype 1b	Genome polyprotein	DP00615	3010
	Hepatitis C virus genotype 2a	Genome polyprotein	DP01031	3033
	Hepatitis GB virus B	Genome polyprotein	DP00674	2864
	Kunjin virus	Genome polyprotein	DP02051	3433
	Murray valley encephalitis virus	Genome polyprotein	DP02212	3434
	West Nile virus	Genome polyprotein	DP02203	3433
			DP00673	3430
	Zika virus	Genome polyprotein	DP01256	3419
Herpesviridae	Epstein-Barr virus	Latent membrane protein 2A	DP01060	118
	Human herpesvirus 1	Envelope glycoprotein B	DP02128	904
		Major viral transcription factor ICP4	DP01305	1298
		TAP transporter inhibitor ICP47	DP02208	88
		Tegument protein VP16	DP02291	490
			DP01642	490
		Thymidine kinase	DP00419	376
	Human herpesvirus 2	Tegument protein VP16	DP00087	490
	Human herpesvirus 8	Kaposi's sarcoma-associated herpes-like virus ORF73 homolog	DP02334	1162
	Human herpesvirus 8	LANA	DP01621	1117
	Human herpesvirus 8 type P	Viral macrophage inflammatory protein 2	DP00685	94
Inovirus	Enterobacteria phage fd	Attachment protein G3P	DP00034	424
Mastadenovirus	Human adenovirus A serotype 12	Early E1A protein	DP01151	266
	Human adenovirus C serotype 2	Early E1A protein	DP01928	289
	Human adenovirus C serotype 5	DNA-binding protein	DP00003	529
		Early E1A protein	DP01150	289
		Pre-protein VI	DP00808	250
Mimivirus	Acanthamoeba polyphaga mimivirus	Probable uracil-DNA glycosylase	DP01481	370

Table S1: Summary of viral protein data

Family	Organism	Protein	Disprot ID	Length
Mimivirus	Acanthamoeba polyphaga mimivirus	Tyrosine–tRNA ligase	DP00726	346
Myoviridae	Enterobacteria phage T4	Baseplate central spike complex protein gp5	DP00284	575
		Deoxycytidylate deaminase	DP00583	193
		Fibritin	DP01616	487
		RNA polymerase-associated protein Gp33	DP00898	112
	Escherichia phage P1	Antitoxin phd	DP00288	73
		Recombination enhancement function protein	DP00932	186
Myoviridae	Escherichia phage P2	Integrase	DP00850	337
Negarnaviricota	Hendra virus	Nucleoprotein	DP00698	532
		Phosphoprotein	DP00700	707
	Human respiratory syncytial virus A	Phosphoprotein	DP00447	241
			DP00895	241
	Influenza A virus	Hemagglutinin	DP00566	566
		Matrix protein 2	DP01016	96
		Nuclear export protein	DP00871	121
	Influenza B virus	Nucleoprotein	DP01405	560
	Lassa virus	RING finger protein Z	DP00820	99
	Measles virus	Nucleoprotein	DP00160	523
			DP00640	525
		Phosphoprotein	DP00133	507
	Nipah virus	Glycoprotein G	DP00686	602
		Nucleoprotein	DP00697	532
		Phosphoprotein	DP00699	709
	Rabies virus	Phosphoprotein	DP01759	297
	Sendai virus	Nucleoprotein	DP00629	524
		Phosphoprotein	DP00939	568
	Vesicular stomatitis Indiana virus	Phosphoprotein	DP01395	265
			DP01394	265
			DP01393	265
			DP01391	265
	Zaire ebolavirus	Hexameric zinc-finger protein VP30	DP00627	288
		Polymerase cofactor VP35	DP00998	340

Table S1: Summary of viral protein data

Family	Organism	Protein	Disprot ID	Length
Nidovirales	Human SARS coronavirus	Nucleoprotein	DP00948	422
Orthohepadnavirus	Hepatitis B virus	Large envelope protein	DP01806	445
Parvovirinae	Adeno-associated virus	Capsid protein VP1	DP01984	733
Picornavirales	Enterovirus D68	VP4	DP00986	69
	Foot-and-mouth disease virus	Genome polyprotein	DP00573	2332
	Mengo encephalomyocarditis virus	Genome polyprotein	DP01129	2293
Podoviridae	Bacillus phage phi29	Capsid assembly scaffolding protein	DP02261	98
	Salmonella phage P22	Transcriptional repressor arc	DP01512	53
Potyviridae	Potato virus Y	Polyprotein	DP01039	594
Reoviridae	Reptilian orthoreovirus	Membrane fusion protein p14	DP01043	125
Retroviridae	Equine infectious anemia virus	Protein Tat	DP00764	78
	HIV-1	Protein Nef	DP00919	208
	HIV-1	Protein Tat	DP01295	72
	HIV-1	Protein Tat	DP01087	101
	HIV-1 subtype B	Envelope glycoprotein gp160	DP00976	856
		Envelope glycoprotein gp160	DP00978	843
		Gag-Pol polyprotein	DP00410	1435
		Gag polyprotein	DP00101	500
		Gag polyprotein	DP00148	512
		Protein Nef	DP01843	206
		Protein Nef	DP00048	206
		Protein Nef	DP00189	206
		Protein Rev	DP00424	116
		Protein Tat	DP00929	86
		Protein Vif	DP00875	192
	HIV-1 subtype C	Protein Tat	DP01003	101
	HIV-1 subtype D	Protein Tat	DP00842	86
	Mason-Pfizer monkey virus	Gag polyprotein	DP01625	657
	Moloney murine leukemia virus	Gag-Pol polyprotein	DP00651	1738

Table S1: Summary of viral protein data

Family	Organism	Protein	Disprot ID	Length
Siphoviridae	Bacillus phage SPP1	39 protein	DP00750	126
	Escherichia phage HK022	Excisionase	DP01013	72
	Escherichia phage lambda	Antitermination protein N	DP00005	107
		DNA-packaging protein FI	DP01336	132
		Head-tail connector protein FII	DP01762	117
		Regulatory protein cro	DP00741	66
Solemoviridae	Southern cowpea mosaic virus	Capsid protein	DP00064	279
Togaviridae	Chikungunya virus	Nonstructural polyprotein	DP01469	2474
			DP01468	2474
			DP01466	2474
		Polyprotein P1234	DP01188	2474
	Semliki forest virus	Structural polyprotein	DP00999	1253
	Sindbis virus subtype Ockelbo	Structural polyprotein	DP00066	1245
Tombusviridae	Carnation mottle virus	Capsid protein	DP02071	348
Tymovirales	Pepino mosaic virus	Coat protein	DP01059	237

Table S2: Summary of non-viral protein data

Family	Organism	Protein	Disprot ID	Length
Chordata	Homo sapiens	60S acidic ribosomal protein P2	DP00793	115
		60S ribosomal protein L4	DP01654	427
		Amyloid-beta precursor protein	DP01280	770
		Anaphase-promoting complex subunit 15	DP01454	121
		Androgen receptor	DP00492	920
		Antigen peptide transporter 2	DP02210	686
		Apoptosis-stimulating of p53 protein 2	DP01164	1128
		ATM interactor	DP01288	823
		ATP-dependent RNA helicase DDX19B	DP01560	479
		Axin-1	DP00959	862
		Beta-adducin	DP00241	726
		Brain acid soluble protein 1	DP00930	227
		Breast cancer type 2 susceptibility protein	DP01869	3418
		Calmodulin regulator protein PCP4	DP00592	62
		cAMP-dependent protein kinase inhibitor alpha	DP00934	76
		C-C motif chemokine 26	DP00696	94
		Cellular tumor antigen p53	DP00086	393
		Cyclin-T1	DP01462	726
		Cysteine protease ATG4B	DP01326	393
		Cystic fibrosis transmembrane conductance regulator	DP00012	1480
		Cytoplasmic protein NCK1	DP01114	377
		DnaJ homolog subfamily C member 24	DP00865	149
		DNA repair protein XRCC4	DP00152	336
		E3 ubiquitin-protein ligase PPP1R11	DP00219	126

Table S2: Summary of non-viral protein data

Family	Organism	Protein	Disprot ID	Length
Chordata	Homo sapiens	E3 ubiquitin-protein ligase XIAP	DP01773	497
		Epidermal growth factor receptor	DP00309	1210
		ETS domain-containing protein Elk-4	DP01329	431
		Eukaryotic initiation factor 4A-III	DP02069	411
		Eukaryotic translation initiation factor 1A, X-chromosomal	DP00903	144
		F-box only protein 4	DP01884	387
		Filamin-binding LIM protein 1	DP01310	373
		Geminin	DP00901	209
		Glycosylphosphatidylinositol-anchored high density lipoprotein-binding protein 1	DP01327	184
		Heterogeneous nuclear ribonucleoprotein F	DP01736	415
		Heterogeneous nuclear ribonucleoproteins A2/B1	DP01109	353
		Homeobox protein Nkx-3.1	DP00683	234
		Hypoxia-inducible factor 1-alpha	DP00262	826
		Immunoglobulin alpha Fc receptor	DP00311	287
		Integrin beta-2	DP01848	769
		Isoform 11 of E3 ubiquitin-protein ligase Mdm2	DP01133	497
		Isoform 2 of Protein max	DP01097	151
		Kinetochore protein NDC80 homolog	DP01576	642
		Kinetochore scaffold 1	DP01269	2342
		Mast/stem cell growth factor receptor Kit	DP02247	976
		M-phase inducer phosphatase 3	DP02126	473

Table S2: Summary of non-viral protein data

Family	Organism	Protein	Disprot ID	Length
Chordata	Homo sapiens	Natriuretic peptides B	DP00551	134
		Neurogenic locus notch homolog protein 1	DP01104	2555
		Nuclear inhibitor of protein phosphatase 1	DP00937	351
		Nuclear pore complex protein Nup133	DP02164	1156
		Nuclear pore complex protein Nup153	DP01799	1475
		Nuclear receptor coactivator 2	DP01880	1464
		Nuclear receptor coactivator 3	DP00343	1424
		Nucleophosmin	DP01474	294
		P antigen family member 5	DP01473	130
		Peroxisome proliferator-activated receptor gamma	DP00718	505
		Polyglutamine-binding protein 1	DP01308	265
		Protein jagged-1	DP00418	1218
		Protein max	DP00084	160
		Protein regulator of cytokinesis 1	DP02316	620
		Protein SMG7	DP01844	1137
		Prothymosin alpha	DP01677	111
		Proto-oncogene c-Fos	DP00078	380
		Ras-related protein Rap-2a	DP00167	183
		Replication protein A 32 kDa subunit	DP01361	270
		Serine/threonine-protein kinase PAK 4	DP01184	591
		Signal recognition particle 19 kDa protein	DP00570	144
		SOSS complex subunit C	DP01943	104
		Stonin-2	DP01368	905
		T-cell surface glycoprotein CD3 gamma chain	DP00508	182
		Thymidylate synthase	DP00073	313
		TP53-regulated inhibitor of apoptosis 1	DP01835	76

Table S2: Summary of non-viral protein data

Family	Organism	Protein	Disprot ID	Length
Chordata	Homo sapiens	Transcription elongation regulator 1	DP01893	1098
		Transcription initiation factor TFIID subunit 6	DP01262	677
		Tyrosine-protein kinase Lck	DP01580	509
		Ubiquitin carboxyl-terminal hydrolase 7	DP00941	1102
	Mus musculus	Amelogenin, X isoform	DP01477	210
		BH3-interacting domain death agonist	DP01661	195
		Dehydrodolichyl diphosphate synthase complex subunit Nus1	DP01304	297
		Dystroglycan	DP00491	893
		Fermitin family homolog 1	DP00655	677
		Mediator of RNA polymerase II transcription subunit 1	DP02151	1575
		Phorbol-12-myristate-13-acetate-induced protein 1	DP01281	103
		Protein BEX1	DP01183	128
		Protein kinase C alpha type	DP01105	672
		Transcription regulator protein BACH2	DP01009	839
		Tumor suppressor ARF	DP00335	169
	Rattus norvegicus	Calcium/calmodulin-dependent protein kinase type 1	DP01958	374
		Calpain-2 catalytic subunit	DP01996	700
		Calpastatin	DP01994	713
		Cyclic AMP-responsive element-binding protein 1	DP00080	341
		Neuroendocrine protein 7B2	DP01557	210
		Olfactory marker protein	DP00279	163
		Rab proteins geranylgeranyltransferase component A 1	DP00458	650

Table S2: Summary of non-viral protein data

Family	Organism	Protein	Disprot ID	Length
Chordata	Rattus norvegicus	Seminal vesicle secretory protein 4	DP00527	112
		Synaptosomal-associated protein 25	DP00068	206
		Vesicle-associated membrane protein 2	DP00622	116
Dikarya	Millerozyma farinosa	Salt-mediated killer protoxin 1	DP00180	222
	Saccharomyces cerevisiae	Acetyl-CoA carboxylase	DP00557	2233
		Autophagy-related protein 13	DP01732	738
		Cold sensitive U2 snRNA suppressor 1	DP01978	436
		DNA-directed RNA polymerases I, II, and III subunit RPABC2	DP00771	155
		DNA topoisomerase 2	DP00076	1428
		Dolichyl-diphosphooligosaccharide–protein glycosyltransferase subunit STT3	DP01195	718
		Eukaryotic initiation factor 4F subunit p150	DP00082	952
		H/ACA ribonucleoprotein complex subunit CBF5	DP02055	483
		Histone H2A.Z-specific chaperone CHZ1	DP01135	153
		Mitochondrial distribution and morphology protein 35	DP02325	86
		Pre-mRNA-splicing factor 18	DP02073	251
		Protein SAN1	DP01136	610
		Protein STE50	DP01515	346
		Regulatory protein ADR1	DP00077	1323
		Ribosome biogenesis protein ERB1	DP00900	807
		Ribosome biogenesis protein NSA1	DP02195	463

Table S2: Summary of non-viral protein data

Family	Organism	Protein	Disprot ID	Length
Dikarya	Saccharomyces cerevisiae	Securin	DP00256	373
		Suppressor protein STM1	DP00994	273
		U6 snRNA-associated Sm-like protein LSm7	DP01261	115
		Ubiquitin-conjugating enzyme E2 1	DP02193	215
		Ubiquitin-like modifier-activating enzyme ATG7	DP02249	630
		UV excision repair protein RAD23	DP01629	398
		Vacuolar-sorting protein SNF8	DP01604	233
	Schizosaccharomyces pombe	YTH domain-containing protein mmi1	DP01975	488
Ecdysozoa	Caenorhabditis elegans	ATP-dependent RNA helicase laf-1	DP01113	708
	Drosophila melanogaster	Chromatin accessibility complex 16kD protein, isoform A	DP00811	140
		FACT complex subunit Ssrp1	DP00720	723
		Transcription initiation factor TFIID subunit 1	DP00081	2129
Streptophyta	Arabidopsis thaliana	Auxin-responsive protein IAA7	DP01121	243
		Calvin cycle protein CP12-2, chloroplastic	DP00534	131
		Dehydrin COR47	DP00657	265

Table S3: Optimized thresholds and accuracy of intrinsic disorder prediction in viral proteins for each predictor analysed. MCC = Matthews correlation coefficient; AUC = area under the receiver operator characteristic curve.

Predictor	MCC	AUC	Specificity	Sensitivity	Threshold
ESpritz.Disprot	0.46	0.85	0.64	0.90	0.29
CSpritz.Long	0.45	0.85	0.57	0.92	0.34
SPOT.Disorder2	0.42	0.84	0.65	0.88	0.13
CSpritz.Short	0.38	0.82	0.67	0.85	0.11
PONDRFIT	0.37	0.80	0.57	0.88	0.50
PONDR.VL3	0.36	0.78	0.56	0.88	0.54
ESpritz.Xray	0.35	0.80	0.58	0.87	0.06
Disprot.vslb	0.35	0.78	0.54	0.88	0.65
PONDR.VSL2	0.35	0.78	0.54	0.88	0.65
IUPRED2.short	0.34	0.79	0.57	0.87	0.47
Disprot.vl3	0.34	0.79	0.53	0.88	0.68
Disprot.vl3h	0.34	0.78	0.55	0.87	0.63
IUPRED2.long	0.33	0.78	0.54	0.87	0.49
Disprot.vl2	0.29	0.76	0.53	0.85	0.57
ESpritz.NMR	0.27	0.74	0.52	0.84	0.36
Disprot.vl2.S	0.24	0.73	0.53	0.81	0.54
PONDR.VLXT	0.22	0.71	0.50	0.80	0.62
Disprot.vl2.C	0.20	0.70	0.57	0.73	0.52
PONDR.XL1	0.15	0.59	0.38	0.82	0.69
Disprot.vl2.V	0.14	0.62	0.29	0.87	0.52
PONDR.CAN	0.11	0.59	0.25	0.88	0.62

Table S4: Optimized thresholds and accuracy of intrinsic disorder prediction in non-viral proteins for each predictor analysed. MCC = Matthews correlation coefficient; AUC = area under the receiver operator characteristic curve.

Predictor	MCC	AUC	Specificity	Sensitivity	Threshold
ESpritz.Disprot	0.36	0.79	0.65	0.78	0.38
ESpritz.Xray	0.35	0.77	0.73	0.70	0.06
SPOT.Disorder2	0.34	0.77	0.70	0.73	0.24
IUPRED2.short	0.33	0.76	0.72	0.69	0.42
IUPRED2.long	0.32	0.76	0.72	0.69	0.47
PONDRFIT	0.32	0.75	0.74	0.67	0.46
PONDR.VSL2	0.30	0.74	0.76	0.63	0.62
PONDR.VL3	0.28	0.74	0.78	0.59	0.51
ESpritz.NMR	0.28	0.73	0.65	0.70	0.39
CSpritz.Long	0.26	0.71	0.83	0.51	0.21
PONDR.VLXT	0.23	0.69	0.70	0.60	0.41
PONDR.XL1	0.18	0.64	0.56	0.67	0.62
PONDR.CAN	0.13	0.60	0.51	0.65	0.34
